# Suppression of Dextran Sulfate Sodium-Induced Colitis in Mice by Radon Inhalation

**DOI:** 10.1155/2012/239617

**Published:** 2012-12-25

**Authors:** Yuichi Nishiyama, Takahiro Kataoka, Keiko Yamato, Takehito Taguchi, Kiyonori Yamaoka

**Affiliations:** Graduate School of Health Sciences, Okayama University, 5-1 Shikata-cho 2-chome, Kita-ku, Okayama-shi, Okayama 700-8558, Japan

## Abstract

The enhanced release of reactive oxygen species from activated neutrophils plays important role in the pathogenesis of inflammatory bowel disease. We previously reported that radon inhalation activates antioxidative functions in various organs of mice. In this study, we examined the protective effects of radon inhalation on dextran sulfate sodium- (DSS) induced colitis in mice which were subjected to DSS for 7 days. Mice were continuously treated with air only (sham) or radon at a concentration of 2000 Bq/m^3^ from a day before DSS administration to the end of colitis induction. In the results, radon inhalation suppressed the elevation of the disease activity index score and histological damage score induced by DSS. Based on the changes in tumor necrosis factor-alpha in plasma and myeloperoxidase activity in the colon, it was shown that radon inhalation suppressed DSS-induced colonic inflammation. Moreover, radon inhalation suppressed lipid peroxidation of the colon induced by DSS. The antioxidant level (superoxide dismutase and total glutathione) in the colon after DSS administration was significantly higher in mice treated with radon than with the sham. These results suggested that radon inhalation suppressed DSS-induced colitis through the enhancement of antioxidative functions in the colon.

## 1. Introduction

 Crohn's disease (CD) and ulcerative colitis (UC) are the two main types of inflammatory bowel disease (IBD), which is chronic inflammatory disease occurring in the intestine. Although their etiologies are still unknown, IBD is now a substantial health problem in many countries. The prevalence of IBD in Japan has been rapidly increasing since the 1980s; in particular, the number of UC patients is much higher than that of CD [1]. In western countries, 1 in 1000 people suffers from IBD [2]. At present, steroidal drugs are commonly used for the treatment of IBD; however, use of these agents is limited due to their adverse effects [3].

 UC is a recurrent inflammatory disorder primarily involving the mucosa and submucosa of the colon. Neutrophil accumulation within epithelial crypts and in the intestinal mucosa directly correlates with clinical disease activity and epithelial injury in UC [4]. Once large numbers of neutrophils and macrophages are activated, these cells enter the injured mucosa of the colon, leading to overproduction of reactive oxygen species (ROS) such as superoxide radical (O_2_^•−^), hydrogen peroxide (H_2_O_2_), and hydroxyl radical (^•^OH) [4]. ROS are highly reactive with cell membranes. When they are generated close to cell membranes, they induce oxidative stress and oxidized membrane phospholipids (lipid peroxidation) and DNA, which could contribute to gene mutation and inflammation, finally causing cancer [5]. 

 It has been reported that low-dose irradiation induces activation of the biological defense system. For example, low-dose X- or *γ*-irradiation attenuates collagen-induced arthritis through suppression of proinflammatory cytokines and autoantibody production, suggesting the enhancement of anti-inflammatory function [6]. In addition, we previously reported that low-dose (0.5 Gy) X- or *γ*-irradiation increases antioxidant substances (e.g., superoxide dismutase (SOD), catalase (CAT), and glutathione (GSH)) and inhibits oxidative damage such as cold-induced brain edema in mice [7]. It is highly possible that low-dose irradiation contributes to the inhibition or treatment of oxidative stress-related diseases such as diabetes [8].

Therapy using radon (^222^Rn), which is volatilized from radon-enriched water and mainly emits *α*-rays, is performed for ROS-related diseases such as osteoarthritis [9] and bronchial asthma [10] in Misasa, Japan and Badgastein, Austria. To clarify the mechanism of the therapy, we investigated the effects of radon inhalation on mice. We previously reported that radon inhalation induced antioxidant substances in many organs, such as the brain, heart, lung, liver, pancreas, kidney, and small intestine of mice [11]. Moreover, we recently demonstrated that radon inhalation inhibited alcohol-induced hepatopathy [12], streptozotocin-induced type-1 diabetes [13], and carrageenan-induced inflammatory paw edema in mice [14]. These findings suggested that radon inhalation has antioxidative and anti-inflammatory effects similar to low-dose X- or *γ*-irradiation; however, there has been no report of the effects of radon inhalation on antioxidative function in the colon. If radon inhalation activates antioxidative functions in the colon, its use is highly likely to be beneficial against colitis; however, there has been no report of the protective effects of radon inhalation against colitis.

 The dextran sulfate sodium (DSS) model of colitis is widely perceived as a good model of experimental colitis because it has similarities to human UC. In this study, we examined the following biochemical and histological parameters to assess the effects of radon inhalation on DSS-induced colitis: myeloperoxidase (MPO), nitric oxide (NO), tumor necrosis factor-alpha (TNF-*α*), SOD, CAT, total glutathione (tGSH), lipid peroxide (LPO) level, and histology.

## 2. Materials and Methods

### 2.1. Animals

Male BALB/c mice (7 weeks of age, approximately 23 g of body weight) were purchased from CLEA Japan Inc. (Tokyo, Japan). They were housed in mice cages (4 mice in each) with wood chip bedding, controlled temperature (20 ± 1°C), and a preset light-dark cycle (12:12 h). They were fed Oriental MF diet (Oriental Yeast Co., Ltd., Tokyo, Japan) and normal drinking water *ad libitum *during all experimental periods. Ethics approval for the experimental design was obtained from the animal experimental committee of Okayama University.

### 2.2. Radon Exposure System

 Radon inhalation was carried out using a radon exposure system developed by the authors [14]. Although the system is a kind of whole-body exposure, it allows the evaluation of the *in vivo* behavior of radon and its physiological effects. Concentration of radon progeny in the exposure box is quite low compared with the concentration of the coexisting radon. The contribution of radon progeny to the experimental animal study results is negligible [15]. 

Although low-dose irradiation activates antioxidative functions in the early stage, these functions gradually come close to the normal level within a few days [16]; however, continuous low-dose irradiation also activates antioxidative functions [17]. In this study, mice continuously inhaled radon at a concentration of 2000 Bq/m^3^ for 8 days except for 1 h during animal care every morning. The radon concentration in the mouse cages was determined by reference to our previous studies. We previously reported that radon inhalation at the concentration of 1000 and 2000 Bq/m^3^ have anti-inflammatory effects and it is better at 2000 Bq/m^3^ than 1000 Bq/m^3^ [18]. The concentration was measured using a radon monitor (CMR-510; Femto-Tech Inc., Carlisle, OH, USA). 

### 2.3. Experimental Procedure

After 7 days of acclimatization, mice were weighed and divided into four groups: sham inhalation only (Control), radon inhalation only (Rn), sham inhalation with DSS administration (Sham+DSS), and radon inhalation with DSS administration (Rn+DSS). Each experimental group consisted of 8 mice. Mice in each group were treated with air only (sham) or radon for 8 days. Mice in Sham+DSS and Rn+DSS groups were induced colitis by replacing normal drinking water with distilled water containing 3% DSS (molecular weight, 4000; Wako Pure Chemical Industry, Co., Ltd., Osaka, Japan) for last 7 days. 

All mice were killed on 7 days after DSS administration by deep ether anesthesia and blood was collected from the heart for NO and TNF-*α* assay in plasma. Plasma was obtained by centrifugation at 3000 ×g for 5 min at 4°C. The colon (from the ileocecal junction to the anal verge) was quickly excised and the length was measured using a vernier caliper. It was then washed with 10 mM phosphate buffer solution (PBS). DSS administration at low molecular weight causes severe mucosal injury in proximal colon [19]; therefore, the proximal colon from the middle was used to assay biochemical activity and histology. These samples were stored at −80°C until use in the biochemical assays or fixed in 10% formalin for histological examination.

### 2.4. Assessment of DAI Score and Weight Gain

To assess the severity of colitis, disease activity index (DAI) score and weight gain were monitored daily. The DAI score was determined based on the methods of Tanaka et al. [20]. Briefly, the DAI score was calculated as the sum of the diarrheal score and the bloody stool score (Table 1). The rate of body weight gain in each mouse was defined by the following formula:

(1) Weight  gain  (%)  ={(weight  each  day)−(weight  at  day  0)weight  at  day  0  }×100

### 2.5. Histology

Tissue samples fixed in 10% formalin were embedded in paraffin. Six micrometer-thick tissue sections were prepared and stained with hematoxylin and eosin (HE) to evaluate mucosal damage. The damage was calculated based on the methods of Araki et al. [21]. Briefly, the damage score was calculated as the sum of three parameters: surface epithelial loss, crypt destruction, and inflammatory cell infiltration into the mucosa (Table 2). 

The sections were also stained with alcian blue to evaluate the presence of goblet cells. We calculated the number of alcian blue-positive goblet cells per unit using ImageJ software (National Institutes of Health, Bethesda, MD, USA).

### 2.6. Biochemical Assays

Mouse colon was homogenized in 1 M Tris-HCl buffer containing 5 mM ethylendiaminetetraacetic acid (EDTA) (pH 7.4) on ice. The homogenate was centrifuged at 12000 ×g for 45 min at 4°C and the supernatant was used to assay the activity of SOD and CAT. SOD activity was measured by the nitroblue tetrazolium (NBT) reduction method [22] using the Wako-SOD test (Wako Pure Chemical Industry, Co., Ltd., Osaka, Japan). Briefly, the extent of inhibition of the reduction in NBT was measured at 560 nm using a spectrophotometer. One unit of enzyme activity was defined as 50% inhibition of NBT reduction.

 CAT activity was measured as the H_2_O_2_ reduction rate at 37°C and was assayed at 240 nm using a spectrophotometer [23]. The assay mixture consisted of 50 *μ*L of 1 M Tris-HCl buffer containing 5 mM EDTA (pH 7.4), 900 *μ*L of 10 mM H_2_O_2_, 30 *μ*L distillated water, and 20 *μ*L colon supernatant. Activity was calculated using a molar extinction coefficient of 7.1 × 10^−3 ^M^−1 ^cm^−1^. CAT activity was measured by the amount of H_2_O_2_ split by CAT at 37°C. The reactions were started by addition of the supernatant.

The tGSH content was measured using the Bioxytech GSH-420 assay kit (OXIS Health Products, Inc., Portland, OR, USA). Briefly, the colon was suspended in 10 mM PBS (pH 7.4), mixed with ice-cold 7.5% trichloroacetic acid solution, and then homogenized on ice. The homogenates were centrifuged at 3000 ×g for 10 min. The supernatant was used for the assay. The tGSH content was measured at 420 nm using a spectrophotometer. This assay is based on the formation of a chromophoric thione, the absorbance of which, measured at 420 nm, is directly proportional to the tGSH concentration.

LPO (malondialdehyde (MDA)) levels were assayed using the Bioxytech LPO-586 assay kit (OXIS Health Products, Inc., Portland, OR, USA). Briefly, the colon was homogenized in 20 mM PBS (pH 7.4) on ice. Before homogenization, 10 *μ*L of 0.5 M butylated hydroxytoluene in acetonitrile was added per 1 mL tissue homogenate. After homogenization, the homogenate was centrifuged at 15000 ×g for 10 min at 4°C and the supernatant was used for the assay. The MDA assay is based on the reaction of a chromogenic reagent, N-methyl-2-phenylidole, with MDA at 45°C. The optical density of the colored products was read at 586 nm using a spectrophotometer.

MPO activity was measured using the Myeloperoxidase (MPO) Colorimetric Activity Assay Kit (BioVision, Inc., Milpitas, CA, USA). Briefly, the colon was homogenized in MPO assay buffer and the homogenate was centrifuged at 13000 ×g for 30 min at 4°C. Supernatants were collected, mixed with MPO assay buffer and MPO substrate, incubated at room temperature for 1 h, and then mixed with tetramethylbenzidine probe. Absorbance was read at 412 nm using a spectrophotometer.

The protein content was measured by the Bradford method [24] using Protein Quantification Kit-Rapid (Dojindo Molecular Technologies, Inc., Kumamoto, Japan).

NO was measured using the Nitrate/Nitrite Colorimetric Assay Kit (Oxford Biomedical Research, Inc., Oxford, MI, USA) according to the manufacturer's recommendations.

TNF-*α* was measured by the enzyme linked immunosorbent assay (ELISA) using the Mouse TNF-*α* ELISA KIT (Shibayagi Co., Ltd., Gunma, Japan) according to the manufacturer's recommendations.

### 2.7. Statistical Analyses

Data are presented as the mean ± standard error of the mean (SEM). The statistical significance of differences between four groups were determined by Tukey's tests. *P* < 0.05 was considered significant.

## 3. Results

### 3.1. General Observation of Colitis

**Figure 1:**
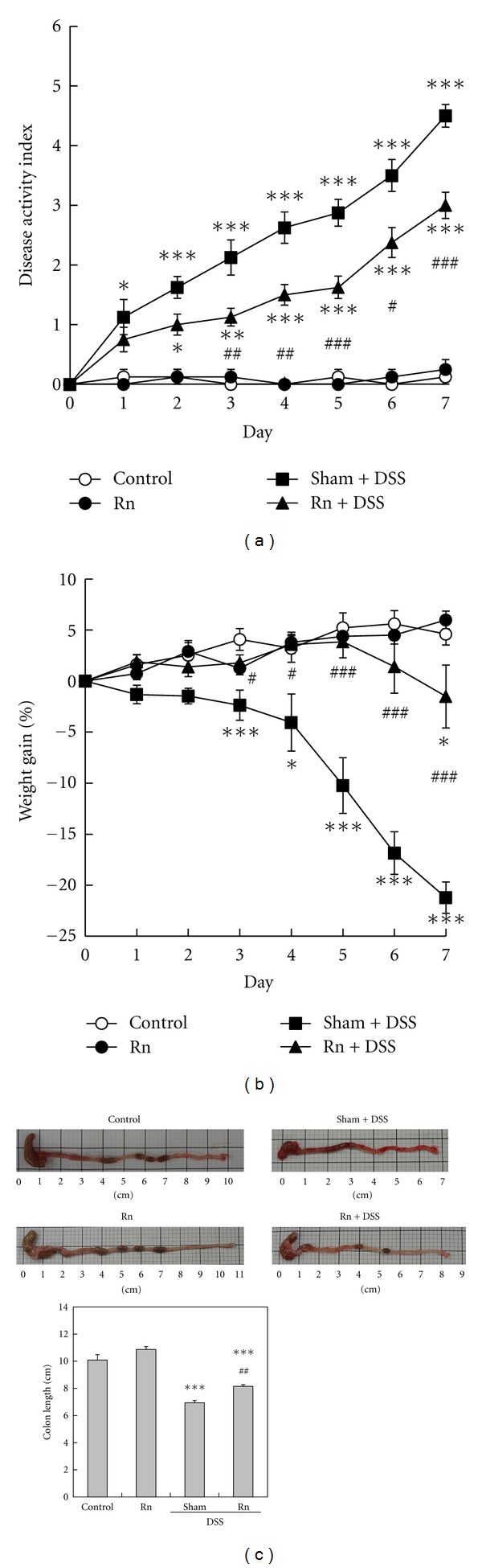
General observation of colitis. (a) Disease activity index score, (b) weight change, and (c) colon length. Each value is the mean ± SEM. The number of mice per experimental point was 8. *P < 0.05, **P < 0.01, and ***P < 0.001 versus control. ^#^ *P* < 0.05, ^##^ *P* < 0.01, and ^###^ *P* < 0.001 versus sham inhalation with DSS administration.

There were no significant differences in the water consumption in each group during colitis induction (data not shown). The DAI score, an indicator of the severity of colitis, was almost 0 in the Control group and Rn group. In Sham+DSS group, the DAI score significantly elevated within 1 day from the beginning of colitis induction. Similarly, in Rn+DSS group, the DAI score significantly elevated within 2 days; however, the DAI scores on day 3 to 7 were significantly lower in Rn+DSS group than in Sham+DSS group (Figure 1(a)). 

The rate of body weight gain in Sham+DSS group significantly began to decrease on day 3 of colitis induction compared with Control group. Similarly, the rate in Rn+DSS group significantly began to decrease on day 7 of colitis induction compared with Control group. However, the rate on day 3 to 7 was significantly higher in Rn+DSS group than in Sham+DSS group (Figure 1(b)).

At the end of the experiment, there were no significant differences in colon length between Rn group and Control group. The colon length in Sham+DSS and Rn+DSS groups was significantly shortened by DSS administration compared with Control group; however, the length was significantly longer in Rn+DSS group than in Sham+DSS group (Figure 1(c)).

### 3.2. Histological Examination

**Figure 2:**
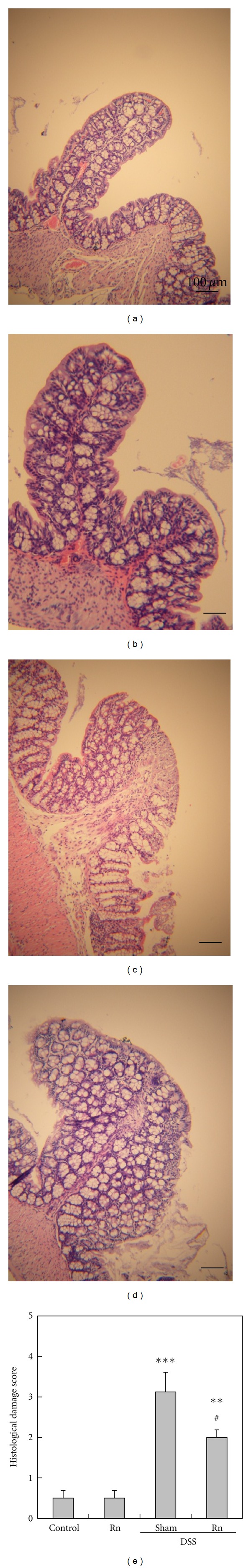
Effects of radon inhalation on mucosal damage after colitis induction. (a) Control, (b) radon inhalation only, (c) sham inhalation with DSS administration, and (d) radon inhalation with DSS administration. All samples were stained with hematoxylin and eosin (HE). Scale bar is 100 *μ*m (×100). (e) Lower histological damage score of mucosa in mice treated with radon inhalation than in mice treated with sham inhalation. Each value is the mean ± SEM. The number of mice for each experiment and significance are the same as in Figure 1.

 In HE-stained sections, morphological changes of the colon were not observed in Rn group compared with Control group. The histological damage score in Sham+DSS and Rn+DSS groups was significantly increased by DSS administration compared with Control group; however, the score was significantly lower in Rn+DSS group than in Sham+DSS group (Figure 2). 

**Figure 3:**
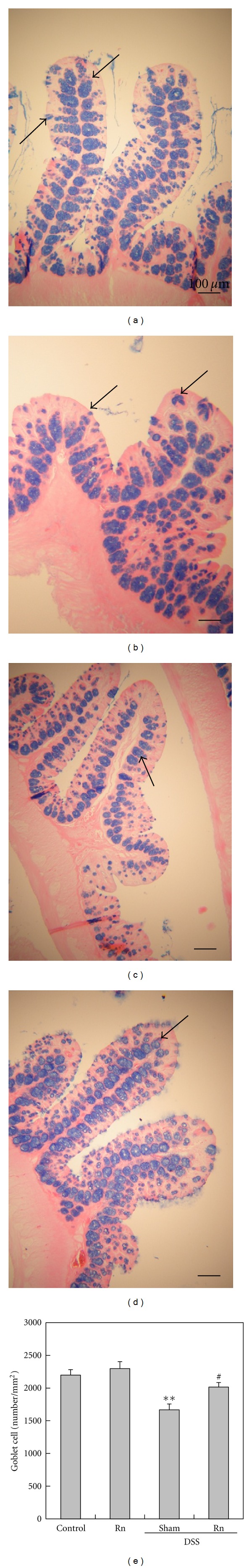
Loss of goblet cells in the colon after colitis induction. (a) Control, (b) radon inhalation only, (c) sham inhalation with DSS administration, and (d) radon inhalation with DSS administration. All samples were stained with alcian blue. Arrow indicates alcian blue-positive goblet cells. Scale bar is 100 *μ*m (×100). (e) Larger number of goblet cells in mice treated with radon inhalation than in mice treated with sham inhalation. Each value is the mean ± SEM. The number of mice for each experiment and significance are the same as in Figure 1.

In alcian blue-stained section, there were no significant differences in the number of goblet cells per unit between Rn group and Control group. The number in Sham+DSS group was significantly decreased by DSS administration compared with Control group; however, the number was significantly larger in Rn+DSS group than in Sham+DSS group (Figure 3).

### 3.3. Effects of Radon Inhalation on Inflammation-Associated Substances and Those after Colitis Induction

**Figure 4:**
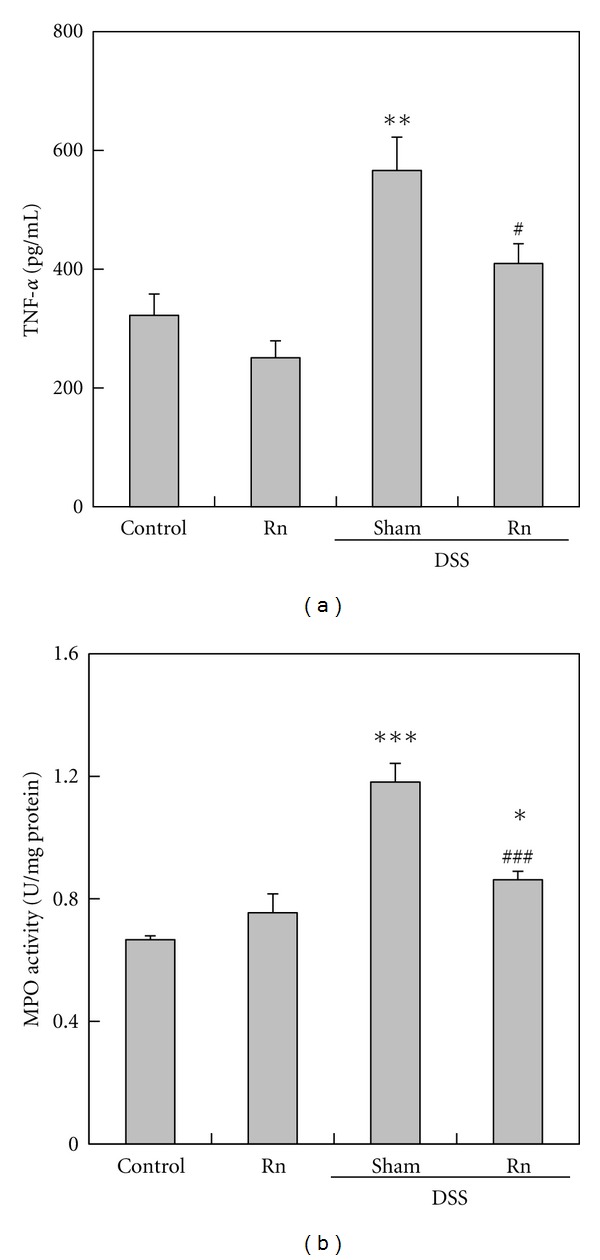
Effects of radon inhalation on inflammation-associated substances after colitis induction. (a) TNF-*α* in plasma and (b) MPO activity in the colon. Each value is the mean ± SEM. The number of mice for each experiment was 7-8. The significance is the same as in Figure 1.

 To assess the effect of radon inhalation on the anti-inflammatory response, TNF-*α* and MPO were assayed following radon inhalation. There were no significant differences in the TNF-*α* level in plasma and the MPO activity in the colon between Rn group and Control group (Figure 4).

Next, we assessed whether radon inhalation suppresses inflammation induced by DSS administration. TNF-*α* level in plasma and MPO activity in the colon in Sham+DSS group were significantly increased by DSS administration compared with Control group; however, they were significantly lower in Rn+DSS group than in Sham+DSS group (Figure 4).

### 3.4. Effects of Radon Inhalation on Antioxidant-Associated Substances and Those after Colitis Induction

**Figure 5:**
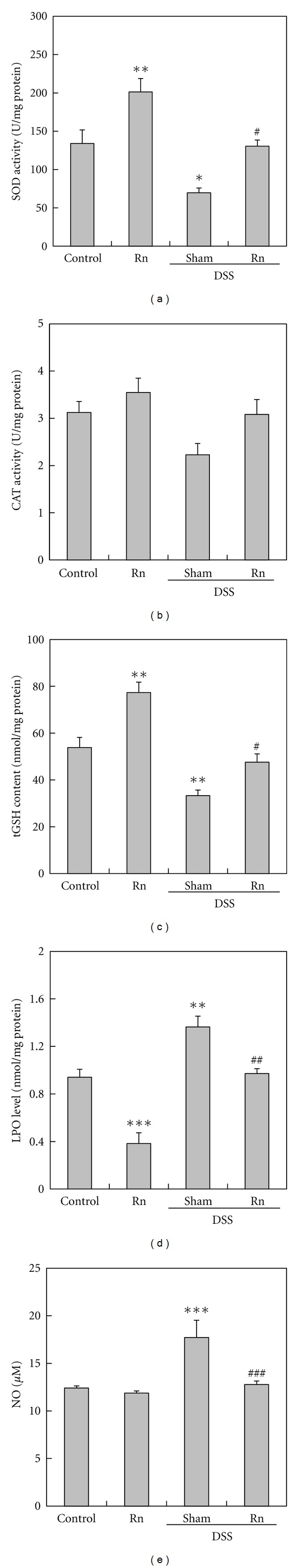
Effects of radon inhalation on antioxidant-associated substances after colitis induction. (a) SOD activity, (b) CAT activity, (c) tGSH content, and (d) LPO level in the colon and (e) NO level in plasma. Each value is the mean ± SEM. The number of mice for each experiment and significance are the same as in Figure 4.

 To assess the effect of radon inhalation on antioxidative functions, SOD, CAT, tGSH, LPO, and NO were assayed following radon inhalation. First, we assessed whether radon inhalation activates antioxidative functions. SOD activity and tGSH content in the colon were significantly higher in Rn group than in Control group (Figures 5(a) and 5(c)). There were no significant differences in CAT activity in the colon and NO level in plasma between Rn group and Control group (Figures 5(b) and 5(e)). LPO level, an indicator of oxidative damage, in the colon was significantly lower in Rn group than in Control group (Figure 5(d)). 

Next, we assessed whether radon inhalation suppresses oxidative damage induced by DSS administration. SOD activity and tGSH content in the colon in Sham+DSS group were significantly decreased by DSS administration compared with Control group; however, they were significantly higher in Rn+DSS group than in Sham+DSS group (Figures 5(a) and 5(c)). LPO level in the colon and NO level in plasma in Sham+DSS group were significantly increased by DSS administration compared with Control group; however, they were significantly lower in Rn+DSS group than in Sham+DSS group (Figures 5(d) and 5(e)).

## 4. Discussion

Radon dissolved in blood entering the gas exchange compartment is transported to many tissues by the blood stream and has stimulus effects [25]. We have demonstrated that radon inhalation activates some biological defense systems such as antioxidative functions and anti-inflammatory functions in various organs in mice; however, the physiological effects of radon inhalation on the colon have never been examined. Therefore, we examined for the first time the effects of radon inhalation on inflammation- and antioxidant-associated substances in the colon. Our results showed that radon inhalation significantly increased antioxidants such as SOD in the colon, indicating the enhancement of antioxidative functions. These findings suggested that radon inhalation may contribute to preventing oxidative stress-related disease in the colon.

 DSS administration induces certain typical symptoms of colitis such as DAI elevation, body weight loss, and a shortened colon [26, 27]. In this study, DSS administration induced the symptoms described above; however, they were clearly suppressed by radon inhalation. These findings indicated that radon inhalation suppressed DSS induced colitis.

 DSS administration causes histological damage such as surface epithelial loss and inflammatory cell filtration into the mucosa [27]. Loss of goblet cells is also one of the pathological features of DSS-induced colitis [27]. In this study, DSS administration induced severe injury to the colon, represented by the elevated histological damage score and decreased goblet cells; however, they were clearly suppressed by radon inhalation. These findings further substantiated the claim that radon inhalation has protective effects on the colon.

Infiltration of neutrophils into colonic tissue causes mucosal damage induced by ROS that are released from activated neutrophils, and this damage further stimulates the infiltration of neutrophils through the induction of proinflammatory cytokines, especially TNF-*α* [4, 28]. TNF-*α* is a primary mediator of the inflammatory response and closely linked to colonic inflammation of UC [29]. MPO is an enzyme found in neutrophils; thus, it is a good marker of inflammation in addition to TNF-*α*. Our results showed that DSS administration induced higher TNF-*α* in plasma and MPO activity in the colon; however, they were significantly decreased by radon inhalation. These findings indicated that radon inhalation suppressed colonic inflammation induced by DSS administration. These findings suggested that radon inhalation reduced mucosal damage and suppressed the further infiltration of neutrophils into the mucosa through the reduction of ROS toxicity.

To further clarify the mechanisms that suppressed colitis, antioxidant-associated substances in the colon and plasma were investigated. Activated neutrophils induced by DSS administration produce ROS, which can cause peroxidation of the cell membrane phospholipids (lipid peroxidation) [4]. In addition, the relation between the severity of colitis and elevated NO synthesis in UC patients and colitis model animals has received much attention [30]. It was reported that TNF-*α* induces the expression of inducible nitric oxide synthetase (iNOS), leading to a steep rise of NO synthesis [31]. In the case of SOD deficiency or increased O_2_^•−^ production, O_2_^•−^ reacts with NO to produce reactive nitrogen species (RNS) such as peroxynitrite (ONOO^−^), which is a highly toxic agent that can cause oxidative/nitrosative stress and functional disorders in the cellular membranes and intracellular proteins [30]. On the other hand, SOD plays an important role in protecting cells from oxidative damage by converting O_2_^•−^ into H_2_O_2_. CAT transforms produced H_2_O_2_ into H_2_O as well as GSH. Many studies have demonstrated that antioxidant agents are beneficial against chemically-induced colitis. For example, SOD administration suppresses DSS-induced colitis by decreasing ROS level in the colon [32], and a similar result was reported in trinitrobenzene sulfonic acid- (TNBS) induced colitis model rats [33]. Another report demonstrated that ROS scavenger edaravone suppresses DSS-induced colitis [21]. Our results in this study were similar to these cases. Antioxidative functions in colon were significantly higher in Rn+DSS group than in Sham+DSS group. These findings suggested that radon inhalation suppressed DSS-induced colitis through the enhancement of antioxidative functions in the colon.

IBD is a chronic and recurrent inflammatory disease. In this study, radon inhalation suppressed acute phase of DSS-induced colitis; however, it is not clear whether radon inhalation is beneficial against chronic inflammation of the colon in IBD. Several studies demonstrated that low-dose irradiation is beneficial against chronic inflammation disease. For example, low-dose X-irradiation inhibits diabetes induced cardiac inflammation in a type-1 diabetes model mouse [34]. In the clinical practices, the therapeutic effects of radon therapy on chronic inflammatory disease such as bronchial asthma have been reported [10]. Radon inhalation has similar effects to low-dose irradiation; therefore, it may contribute to the treatment of chronic inflammation in IBD. Further study is required in this point.

Immune factor also plays important role in the pathogenesis of colitis. Anezaki et al. reported that interleukin-8 (IL-8) level is closely correlated to MPO levels in the inflamed mucosa of UC [35]. In UC patients, IL-8 mRNA was found mainly in macrophages, and also in neutrophils and colonic epithelial cells [36]. Moreover, increased production of IL-8 peptides and expression of IL-8 mRNA are observed in the inflamed mucosa of UC [37]. These findings may suggest that IL-8 is a neutrophil-activating substance to release ROS from neutrophil. In this study, the effects of radon inhalation on the intestinal immunity were not examined. It was not clear whether radon inhalation suppressed DSS induced colitis through the direct inhibition of neutrophil activation. More detailed studies from the viewpoint of immune factor in intestinal mucosa will be necessary for further clarification of the mechanism that radon inhalation suppressed DSS-induced colitis. 

Radon has been recognized as a cause of lung cancer [38]; however, most of the doses to the lung come from its short-lived progeny and not from radon itself. Moreover, lifestyle influences such as smoking is larger than radon exposure [39]. In this study, the influence of radon progeny to mice is negligible [15]. Absorbed dose into the lungs and colon of mice under our experimental condition were estimated both to be within the range of 0.67−1.2 *μ*Gy according to our previous report [25]. The International Commission on Radiological Protection (ICRP) expresses an opinion that a significant cancer risk associated with exposure under 100 mSv has not been demonstrated [40]. Based on the recommendations of the ICRP, the risks associated with exposure to radon under our experimental condition are low. In fact, adverse or negative effects of radon therapy have not been reported in the past. 

## 5. Conclusion

In conclusion, radon inhalation activated antioxidative functions and suppressed oxidative damage and inflammation in the colon induced by DSS administration. Beneficial effects of radon inhalation in the treatment of IBD patients are strongly expected. At present, steroidal drugs which have adverse effects are commonly used for the treatment of IBD. The introduction of radon inhalation for the treatment of IBD may contribute to reduce the drug dosage and its adverse effects. The data presented in this study provide a substantial basis for future studies aimed at assessing new radon-based therapies for the treatment of IBD in humans. 

In this study, we demonstrated that continuous radon inhalation suppressed DSS-induced colitis; however, such long term inhalation is unsuitable for medical treatment. We aim to research the optimum condition for achieving the maximal effect under short time inhalation. In the future, more detailed studies of the following points will be necessary for further application of radon therapy to medical treatment: further clarification of the mechanisms of the health effects, research into new indications, determination of the optimum condition for achieving the maximal effect, and assessment of concomitant effects with drugs. 

## Figures and Tables

**Table 1:** Disease activity index (DAI) scoring system.

Score	Diarrheal stool score	Bloody stool score
0	Normal stool	Normal colored stool
1	Mildly soft stool	Brown stool
2	Very soft stool	Reddish stool
3	Watery stool	Bloody stool

The sum of the scores of two parameters was defined as the DAI score.

**Table 2:** Histological damage scoring system.

Parameters	Score	Histological features
(i) Surface epithelial loss	0	No change
(ii) Crypt destruction	1	Localized and mild
	2	Localized and moderate
(iii) Inflammatory cell infiltration into the mucosa	3	Extensive and moderate
	4	Extensive and severe

The sum of the scores of three parameters was defined as the mucosal damage score.

## Abbreviations

CAT: Catalase
CD: Crohn's disease
DAI: Disease activity index
DNA: Deoxyribonucleic acid
DSS: Dextran sulfate sodium
EDTA: Ethylendiaminetetraacetic acid
ELISA: Enzyme linked immunosorbent assay
GSH: Glutathione
HE: Hematoxylin-eosin
H_2_O_2_: Hydrogen peroxide
IBD: Inflammatory bowel disease
ICRP: International Commission on Radiological Protection
IL-8: Interleukin-8
iNOS: Inducible nitric oxide synthetase
LPO: Lipid peroxide
MDA: Malondialdehyde
MPO: Myeloperoxidase
mRNA: Messenger ribonucleic acid
NBT: Nitroblue tetrazolium
NO: Nitric oxide
O_2_^•−^: Superoxide anion radical
^•^OH: Hydroxyl radical
ONOO^−^: Peroxynitrite
PBS: Phosphate buffer saline
RNS: Reactive nitrogen species
ROS: Reactive oxygen species
SEM: Standard error of mean
SOD: Superoxide dismutase
tGSH: Total glutathione
TNBS: Trinitrobenzene sulfonic acid
TNF-*α*: Tumor necrosis factor-alpha
UC: Ulcerative colitis.
